# Extracellular Vesicles as a New Therapeutic Entity

**DOI:** 10.3390/life13061235

**Published:** 2023-05-24

**Authors:** Yong Weon Yi

**Affiliations:** 1 Department of Biochemistry, College of Medicine, Dankook University, Cheonan-si 31116, Chungcheongnam-do, Republic of Korea; yongweon_yi@dankook.ac.kr; 2 Multidrug-resistant Refractory Cancer Convergence Research Center (MRCRC), Dankook University, Cheonan-si 31116, Chungcheongnam-do, Republic of Korea

Collectively, extracellular vesicles (EVs) refer to vesicular entities secreted by live cells, including microvesicles, exosomes, and apoptotic bodies [1,2,3] It has been now well established that EVs play important roles in intercellular communication and the physiological regulation of cellular functions in both healthy and disease conditions [4,5]. More importantly, growing evidence supports the notion that EVs have potential as a therapeutics and drug delivery tool with compatible tolerance and excellent delivery efficacy [6].

Research for EVs is a rapidly growing field in current biology. The number of publications peaked to more than 8000 papers (PubMed search with keywords exosomes OR exosome OR “extracellular vesicles” OR “extracellular vesicle” at 26 April 2023) in 2022 from more than 3000 papers in 2018 [3]. This Special Issue, entitled “The Therapeutic Applications of Extracellular Vesicles,” features a collection of three research articles and three reviews on the latest research focused on the application of EVs as new therapeutics. The papers in this Special Issue cover a range of topics, from bovine milk exosomes as an anticancer agent to EVs derived from mesenchymal stem cells (MSCs) for attenuating brain injury in bacteria-induced meningitis in newborn rats.

MSC-derived EVs (MSC-EVs) are now well established as potential therapeutics due to their regenerative potential and immune modulation [3,7,8,9,10,11]. A research article reported a novel therapeutic effect of MSC-EVs on meningitis in newborn rats induced by *Escherichia coli* (*E. coli*) [12]. Intra-cerebroventricular transplantation of MSC-EVs attenuated brain cell death, reactive gliosis, and inflammation including the number of active microglia and the production of inflammatory cytokines, while there was no significant effect on bacterial growth. These results suggest MSC-EVs as a potential combination partner with antibiotics for the treatment of neonatal meningitis caused by bacterial infection.

EVs from the sera of patients have potential diagnostic and prognostic value [13,14,15] and a commercial diagnostic kit for prostate cancer is already available [15]. An article reported analyses of serum-derived EVs from patients with severe liver injury chronic hepatitis B (CHB) and patients with hepatitis B virus (HBV)-associated decompensated cirrhosis (DeCi) [16]. Interestingly, the highest EV concentration was found in the serum of patients with severe liver injury-CHB. The miRNA-seq revealed that miR-172-5p and miR-1285-5p in the serum EVs of patients were markedly downregulated and had predictive values for the progression of the normal controls to severe liver injury-CHB. In addition, miR-335-5p, which was downregulated in the DeCi group compared to the severe liver injury-CHB group, added a predictive accuracy of the progression of severe livery injury-CHB to DeCi.

The functionalization of EV surfaces has been studied for improving EV characteristics for tissue targeting, in vivo stability, real-time tracking, the loading and sustained release of drugs, in vivo tolerance, etc. [6,17,18]. A paper in this issue described the therapeutic use of folic acid (FA)-functionalized bovine milk exosomes for treating breast cancer [19]. Since the severe toxicity of paclitaxel is derived from the adjuvant Cremophor EL^®^ in ethanol [20], FA-functionalized exosomes were developed for the oral delivery of paclitaxel in combination with 5-fluorouracil (5-FU). The advantages of these exosomes are sustained drug release up to 48 h, a decrease in IC value, a higher apoptotic index, and better control of cell migration [19]. In addition, milk exosomes have the advantages of good oral bioavailability with good biocompatibility, and an ability to cross the cellular barriers and escape from the reticuloendothelial system [21]. Milk-derived exosomes are a potential drug delivery vehicle due to their biocompatibility, stability, cost-effectiveness, and abundance [22,23]. With these advantages, functionalization with FA achieves tumor targeting because folate receptor is a tumor-associated antigen [24].

The three-dimensional (3D) culture of MSCs has been reported to enhance the yield of exosome production from MSCs [25,26,27,28]. A review paper in this issue [29] summarized the recent advances in the application of 3D culture techniques for MSCs and the therapeutic application of MSC-exosomes from 3D spheroid culture of MSCs to enhance cartilage regeneration for osteoarthritis. Various 3D culture techniques were overviewed and studies which conducted the chondrogenic effects of 3D cultured cells were discussed. Additionally, a recent trend using 3D-bioprinting technology for spheroids was reviewed.

Cargo in EVs includes various proteins, nucleic acids, lipids, and metabolites. Noncoding RNAs such as microRNAs (miRNAs) and long noncoding RNAs regulate diverse cellular processes. As mentioned earlier, miRNAs carried by EVs derived from patients’ sera are a good diagnostic biomarker [13,14,15]. A review paper summarized the potential of EV-miRNAs as molecular markers for chronic respiratory diseases (CRDs) [30]. CRDs include idiopathic pulmonary fibrosis (IPF), chronic obstructive pulmonary disease (COPD), asthma, and sleep disorders [31]. Although genetic predisposition is the main cause of CRDs, environmental risk factors such as allergens, tobacco smoke, occupational exposure, and air pollution exacerbate CRDs [32]. A diverse set of miRNA profiles have been reported to be correlated with CRDs [30]. Although no miRNA has been established as a biomarker for specific types of CRDs, the continuous progress of EV research may render EV-miRNAs diagnostic or prognostic markers and therapeutic interventions for lung diseases.

The coronavirus infection disease 2019 (COVID-19) pandemic deeply impacts science our and daily lives. Interestingly, EVs derived from cells infected by severe acute respiratory syndrome coronavirus type 2 (SARS-CoV-2) contain viral particles and other cargo molecules that may affect the host immune system and facilitate viral infection [33,34]. A review paper overviewed the biology of SARS-CoV-2 infection and the roles of EVs in viral infection [35]. In addition, strategies using EVs as a therapeutic entity and as a potential therapeutic target were also discussed. Since EVs can carry antigens on their surface, EV-based vaccines are also another potential tool to prevent viral infection, including COVID-19.

The intention of this Special Issue is to provide a unique international forum covering a broad description of results surrounding the current advances in EV-based therapeutics. Hopefully, this Special Issue will inspire further research on the clinical development of EVs as a new therapeutic entity.
